# Retroperitoneal Castleman's tumor and paraneoplastic pemphigus: report of a case and review of the literature

**DOI:** 10.1186/1477-7819-5-45

**Published:** 2007-04-28

**Authors:** Charalambos Menenakos, Chris Braumann, Jens Hartmann, Christoph A Jacobi

**Affiliations:** 1 Department of General, Visceral, Vascular and Thoracic Surgery, Universitaetsmedizin Berlin, Charité Campus Mitte, Berlin, Germany

## Abstract

**Background:**

Castleman's disease is a rare lymphoproliferative syndrome. Its etiology and pathogenesis are unclear. The disease can be occasionally associated with a paraneoplastic pemphigus (PNP), an autoimmune mucocutaneous disorder commonly seen in neoplasms of lymphocytic origin.

**Case presentation:**

We present a case of a 63-year old male patient who was referred for surgical treatment of a lately diagnosed retroperitoneal pelvic mass. The patient had been already treated for two years due to progressive diffuse cutaneous lesions histologically consistent with lichen ruber verucosus and pemphigus vulgaris. Intraoperatively a highly vascularized solid mass occupying the small pelvis was resected after meticulous vascular ligation and hemostasis. After surgery and following immunosuppressive treatment a clear remission of the skin lesions was observed.

**Conclusion:**

Castleman's tumor should be always suspected when a retroperitoneal mass is combined with PNP. In a review of the literature we found 37 additional cases. Complete surgical resection of the tumor can be curative in most of the cases.

## Background

Castleman's disease is a rare lymphoproliferative syndrome which was first described in 1956 [1]. The etiology and pathogenesis of this entity is still unclear. The hyaline vascular type frequently appears as a benign isolated mediastinal or rarely retroperitoneal mass, which does not recur after curative surgical excision. The plasma-cell type is associated with constitutional symptoms, multicentric lymphnode involvement, lymphoma development and autoimmune disease like clinical and laboratory abnormalities, including paraneoplastic pemphigus (PNP) [2].

PNP is an autoimmune mucocutaneous disorder associated with neoplasms of lymphocytic origin, among them with Castleman disease [3]. PNP can mimic a variety of dermatological diseases including pemphigus vulgaris, erythema multiforme, erosive lichen planus and acute lupus erythematosus, so that the correct diagnosis is often delayed. The role of the surgeon is essential as resection of the tumor could be curative in many cases.

We report a case of a patient with a retroperitoneal Castleman's tumor and paraneoplastic pemphigus that improved after tumor resection. A review of the literature is additionally presented.

## Case presentation

A 63-year-old male patient was referred to our Surgical Department from the Department of Dermatology with the diagnosis of a large retroperitoneal pelvic tumor for further treatment. The mass was morphologically consistent with a Castleman's tumor. Patient's symptoms had begun two years earlier with rapidly progressive diffuse cutaneous lesions all over the trunk and extremities as well as oral lesions with the form of erythematous plaques and superficial hyperceratosis. Erosive mucositis with lichenoid inflammation affecting the oral and penis glance mucosa as well as dystrophic nails associated with periungular erosions and onychorrexis had been observed. The patient had been initially treated with local corticosteroids and acitretin (Neotigason^®^) but skin lesions had remained stable or had shown a mild shortlasting remission only. The histological examination of the lesions was consistent with lichen ruber verucosus and pemphigus vulgaris.

One month before admission the patient was intravenously treated for an atypical pneumonia and alveolitis with Rocephin and Prednisolon (100 mg for 3 days). As skin lesions had shown no signs of remission, further diagnostics was initiated in order to determine a possible paraneoplastic nature of skin pathology.

**Figure 1:**
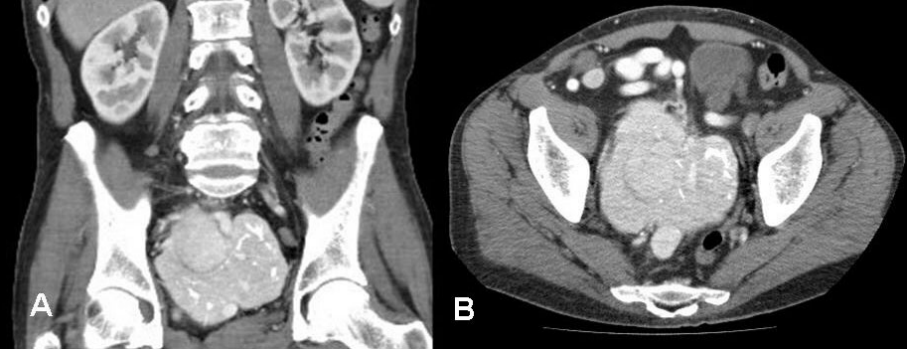
A). Preoperative CT scan of the abdomen showing a highly vascularized retroperitoneal tumor measuring 10 × 9.2 cm with intratumor calcifications. Right ureter dilatation (grade II), (coronal multiplanar reformation, MPR) B). The same tumor in axial orientation. No other tumor localization in the abdomen nor enlarged lymph nodes were detected.

A subsequently performed contrast-enhanced multislice CT demonstrated a highly vascularized retroperitoneal mass (10.3 × 9.2 cm) with significant contrast enhancement consistent with a sarcoma or a Castleman's tumor (Figure 1a and 1b). The tumor filled the whole lower pelvis without any signs of adjacent organs' invasion though.

On physical examination besides the above mentioned diffuse erosive muscositis with lichenoid inflammation, no lymphadenopathy or organomegaly was noted. Preoperative examination of the complete blood counts revealed the following results: hematocrite 41%, white blood cells 11.0/nl (80% neutrophils), PTL 562 k/m, PT 90% (INR 1.07).

Following thorough preoperative diagnostics the patient was submitted to laparotomy for resection of the tumor. After catheterisation of both ureters with "pig-tail" catheters a midline laparotomy was performed. Entrance into the peritoneal cavity revealed a highly vascularized solid mass occupying the small pelvis extending to the right iliac fossa.

After careful dissection of the right iliac vessels and right ureter off the mass, complete excision of the bulky tumor could be performed. Special attention was given to the meticulous ligation of the tumor vessels, mainly collaterals coming from the internal iliac artery, which was separately ligated.

After surgery the patient was treated for four days on the ICU. Postoperative course was uneventful, so that the patient could be referred to the Dermatology Department for further treatment of his skin lesions. A complete evaluation of the patient's immune status was performed. Main results are as following: lymphocytes 5%, CD3+ 61% (reference value 59.4–84.6), CD3, CD4/CD3, CD8 0.91 % (0.9–3.6), CD3/CD19 12% (6.4–22.6), CD3/CD4 30 % (28.5–60.5), CD3/CD8 33 % (11.1–38.3), CD3/CD16+56 23% (5.6–30.9). Anti-Desmoglein 1-Abs were 59.96 (>20 positive), anti-Desmoglein 3-Abs 48.25 (>20 positive), BP 180-Abs 6.25 U/ml (>9 positive). Skin Abs were negative, but circulating IgG antibodies reactive with rat urinary bladder epithelia surfaces were detected positive (diagnostic of paraneoplastic pemphigus). New histological examination of biopsies taken from skin lesions, extremities, and the oral mucosa were consistent with the diagnosis of lichen ruber and highly suggestive of a pemphigus. The latest diagnosis was confirmed with direct immunofluorescence, which showed a high intracellular deposition of anti-IgG (antibodies against desmosomes) in the whole epidermis. Histological examination of the mass revealed a Castleman tumor of hyaline vascular type locally in sano removed.

One month after surgery the patient was newly referred to us with a postoperative 15 × 11 cm pelvic abscess, which was percutaneously drained under CT control. Culture of the drained pus was positive for staphylococcus aureus (MRSA) and an intravenous treatment was initiated (linezolid and flucloxacillin). Percutaneous drainage was repeated two weeks later so that a rest fluid around the right psoas muscle could be successfully evacuated. Due to progression of the painful skin lesions an immunosuppressive treatment was initiated with cyclophosphamide and urbason (every two weeks) and cortison daily followed by a per os immunosuppression protocol with cyclophosphamide and endoxan.

A dramatic improvement of the skin lesions could be seen in a period of three weeks' time. Following a removal of the left ureter catheter and change of the right one, the patient could be released in a good condition with almost complete remission of the mucocutaneous lesions four weeks later (2 months after initial surgery).

## Discussion

Castleman's tumors are neoplasms of lymphatic origin, also known as giant lymph node hyperplasia or benign giant lymphoma. Histologically these tumors can be classified into 3 types: a) Hyaline-vascular type (80–90%), b) plasma cell type (10–20 %), and c) intermediate types [4]. The most common location of the tumor is the mediastinum (60 – 70 %). Abdominal forms are rare (10–17 %) and the majority is being retroperitoneal. In our patient a hyaline-vascular type with retroperitoneal location was diagnosed. Besides the localized form multicentral variants with and aggressive clinical course, systemic symptoms, organomegaly and neoplastic transformation have been reported. Castleman's disease has been associated with a very high incidence of autoimmune phenomena such as cytopenia, peripheral neuropathy, systemic lupus erythematosus, Sjögren's syndrome, and myasthenia gravis [5].

PNP is a clinically, histologically and immunologically distinct autoimmune mucocutaneous disease. This entity was first described by Anhalt et al. [6]. A variety of neoplasms have been reported in PNP. These comprise haematological diseases including Non-Hodgkin Lymphoma, chronic lymphocytic leukaemia, and Waldenstroems's macroglobulinaemia. More rarely there is an association with solid tumors such as sarcoma, bronchial carcinoma, colonic dysplasia, Castleman's disease and thymoma [7,8]. Castleman's tumor has been found in approximately 10 % of PNP patients [4]. The distinctive clinical findings in PNP include severe painful oral erosions and ulcerations with hemorrhagic crusting of the lips and polymorphous skin lesions resembling erythema multiforme, pemphigus vulgaris (PV) or lichen planus pemphigoides [9]. These clinical findings were observed in our patient. Cases of PNP with pulmonary involvement resulting in respiratory failure have been also reported [9]. So it can not be excluded that the atypical pneumonia and alveolitis of our patient could be related to the Castleman's tumor.

So far, it is unclear why Castleman's tumors are related to the pathogenesis of PNP. One hypothesis is that Castleman's tumors' proteins function as antigens and induce the production of autoantibodies in patients with paraneoplastic pemphigus. Meanwhile a general propensity for immune dysregulation has been proposed [4].

Several cases of association between pemphigus and Castleman's tumor have been reported. In a review of the english language literature we found 37 additional cases [1-5,7-22]. In our case the diagnosis of paraneoplastic pemphigus was retrospective. Initially it was described as lichen planus in association with pemphigus. Detection of the Castleman's tumor established the clinical correlation.

Treatment of the disease depends mainly on the histological type and the clinical symptoms [23]. Surgery arises as the golden standard for the treatment of localized disease with curative results in most of the cases. High doses of corticosteroids, radiation, chemotherapy and immunosuppressive therapy have been used as additional therapeutic modalities. It should be however pointed out that an PNP immunosuppressive treatment alone is ineffective without treatment of the underlying neoplasm. On the contrary, a complete remission of the skin lesions has been observed once the tumor has been removed obviating the need for further immunosuppressive treatment [3,7]. This observation emphasizes on the role of surgeons in the treatment of the syndrome with an early and complete excision of the tumor in compliance with principles of surgical oncology. We have to point out that complete surgical resection, although curative, is in some cases precluded due to hypervascularity of the tumor or invasion of adjacent structures [1]. Meticulous hemostasis, protection of major vessels and adjacent organs and complete removal of the tumor when possible are basic surgical principles that should be kept when operating these tumors.

In the present case, although there was an initial deterioration of skin lesions after tumor resection, the lichenoid lesions dramatically improved after the initiation of immunosuppressive therapy, leaving a postinflammatory hyperpigmentation.

## Conclusion

Castleman's disease is a diagnosis, which should be considered in the presence of a hypervascular tumor, whatever its localization. Additionally PNP should be always considered when investigating atypical mucocutaneous lesions such as erosive lichen planus or pemphigus vulgaris non-responding to medication. In these cases a prompt search for an underlying tumor should be initiated. Complete surgical resection of the tumor can be curative in most of the cases.

## Competing interests

The author(s) declare that they have no competing interests.

## Authors' contributions

**CM **Member of operative team which performed the operation, Section "Discussion"

**CB **Review of the literature, Section "Discussion"

**JH **Collection of information about patient's previous dermatological history, writing of the section "Case report"

**CAJ **Member of operative team which performed the operation, final form of the manuscript
